# Bis[2-(Methacryloyloxy) Ethyl] Phosphate as a Primer for Enamel and Dentine

**DOI:** 10.1177/00220345211023477

**Published:** 2021-07-08

**Authors:** R. Alkattan, G. Koller, S. Banerji, S. Deb

**Affiliations:** 1 Centre for Oral, Clinical and Translational Sciences, Faculty of Dentistry, Oral and Craniofacial Sciences, King’s College London, London, UK; 2 Department of Restorative Dental Science, King Saud Bin Abdulaziz University for Health Sciences, Riyadh, Saudi Arabia; 3 Centre for Host Microbiome Interactions, King’s College London Dental Institute at Guy’s Hospital, King’s Health Partners, London, UK; 4 London Centre for Nanotechnology, London, UK; 5 Department of Prosthodontics, University of Melbourne, Melbourne, Australia

**Keywords:** adhesives, dental bonding, matrix metalloproteinases, phosphoric acid esters, dental etching, polymerization

## Abstract

Dental resin composites are commonly used in the restorative management of teeth via adhesive bonding, which has evolved significantly over the past few decades. Although current self-etch bonding systems decrease the number of clinical steps, the acidic functional monomers employed exhibit a limited extent of demineralization of enamel in comparison to phosphoric acid etchants, and the resultant superficial ionic interactions are prone to hydrolysis. This study evaluates the etching of primers constituted with bis[2-(methacryloyloxy) ethyl] phosphate (BMEP) of dental hard tissue, interfacial characteristics, and inhibition of endogenous enzymes. We examine the incorporation of 2 concentrations of BMEP in the formulation of experimental primers used with a hydrophobic adhesive to constitute a 2-step self-etching bonding system and compare to a commercial 10–methacryloyloxydecyl dihydrogen phosphate (10-MDP)–containing system. The interaction of the primer with enamel and dentine was characterized using scanning electron, confocal laser scanning, and Raman microscopy while the polymerization reaction between the BMEP primers and hydroxyapatite was evaluated by Fourier-transform infrared spectroscopy. The inhibitory effect against matrix metalloproteinase (MMP) enzymes of these primers was studied and percentage of inhibition analyzed using 1-way analysis of variance and Tukey’s post hoc test (*P* < 0.05). Results of the scanning electron microscopy micrographs demonstrated potent etching of both enamel and dentine with the formation of longer resin tags with BMEP primers compared to the 10-MDP–based system. The BMEP polymerized on interaction with pure hydroxyapatite in the dark, while the 10-MDP primer exhibited the formation of salts. Furthermore, BMEP primers were able to inhibit MMP activity in a dose-dependent manner. BMEP could be used as a self-etching primer on enamel and dentine, and the high degree of polymerization in the presence of hydroxyapatite can contribute to an increased quality of the resin polymer network, prompting resistance to gelatinolytic and collagenolytic degradation.

## Introduction

Advances in development of adhesive systems have hugely enhanced the scope of dental resin composites for restoration of teeth. Etch-and-rinse adhesives (ERAs) have been successful when enamel margins are involved in restorations but are associated with overetching of dentine, moisture sensitivity, and desiccation (Perdigão 2020). Self-etching adhesives (SEAs) were primarily developed to overcome marginal leakage, recurrence of secondary caries, and moisture sensitivity of ERAs and to reduce the number of clinical steps. They also decrease discrepancy between etching and resin infiltration associated with ERAs, thereby creating a more homogeneous hybrid layer (Breschi et al. 2004). However, the main drawback of SEAs is that bond strengths to enamel are inferior due to lower acidity of self-etch monomers in comparison to phosphoric acid solutions and hence are unable to etch enamel properly, especially with milder SEAs (De Munck et al. 2005; Hass et al. 2017). The typical appearance of a honeycomb pattern of exposed enamel prisms and formation of distinct resin tags in dentine have only been reported with stronger SEAs (Wang et al. 2017; Hoshika et al. 2018). Nonetheless, SEAs offer the advantage of chemical interactions with hydroxyapatite (HA) in addition to micromechanical retention (Yoshida et al. 2004; Yoshihara et al. 2018).

Functional monomers in SEAs typically comprise at least one polymerizable group and a functional group responsible for wetting and demineralizing the tooth tissue and promoting interaction with apatites. Frequently used in SEAs due to its strong binding affinity to tooth apatite arising from interaction of the phosphate group with calcium ions, 10–methacryloyloxydecyl dihydrogen phosphate (10-MDP) forms an ionic bond with enamel and dentine (Yoshida et al. 2004); 10-MDP is also hydrophobic in nature due to the long alkyl chain and resistant to hydrolysis (Van Landuyt et al. 2007). However, 10-MDP–based SEAs superficially demineralize enamel and form shallow dentine resin tags approximately 1 µm deep, indicating the somewhat superficial interactions in 10-MDP–Ca salts (Wang et al. 2017).

While acidic monomers in bonding systems penetrate and demineralize dentine, the adhesive is usually unable to reach the full depth of demineralized dentine that often leads to mineral-depleted collagen at the base of the hybrid layer with no protection from the polymerized resin. This denuded collagen then becomes prone to degradation through slow and gradual release of collagenolytic enzymes from the dentine matrix (Hashimoto et al. 2003). Any unreacted acidic monomers remaining within dentine tubules cause continued etching that would have otherwise been neutralized by surrounding minerals in dentine (Wang and Spencer 2005). To overcome the effects of collagenolytic enzymes, matrix metalloproteinase (MMP) inhibitors, particularly chlorhexidine (CHX), is able to preserve the collagen matrix in hybrid layers, thereby decreasing degradation of resin-dentine bonds (Carrilho et al. 2007; Breschi et al. 2010). However, as CHX is water soluble, it may leach out and its cationic binding reversed (Mazzoni et al. 2015). Since MMP inhibition of CHX is related to calcium chelation, increased calcium concentrations may mitigate the inhibitory effect on MMPs (Zhou et al. 2011). Hence, more permanent MMP inhibitors have been considered to cross-link collagen fibrils, making them more resistant to proteolytic degradation (Bedran-Russo et al. 2014).

Bis[2-(methacryloyloxy) ethyl] phosphate (BMEP) is a monomer with a centrally located phosphate group flanked by 2 polymerizable methacrylate groups that is able to undergo spontaneous polymerization with a high degree of conversion in the presence of HA (Zhang and Wang 2012a, 2012b; Zhang et al. 2013; Liu et al. 2016). Furthermore, the hydrophilicity and short carbon chains in BMEP may enhance wetting, while its small size and dimethacrylate groups could enable cross-linking and improve copolymerization with comonomers in the adhesive system (Feitosa et al. 2014; Wang et al. 2017). However, whether this will contribute to a stable hybrid layer and protect the collagen from the action of endogenous MMPs has not been established.

This study examines incorporation of BMEP in experimental primers used with a hydrophobic adhesive to constitute a 2-step self-etching bonding system for use on both enamel and dentine. The hypothesis is that the hydrophilic BMEP could enhance resin infiltration and the dimethacrylate groups enable cross-linking. The extent of etching of BMEP-based primers on enamel and dentine and inhibition of MMP enzymes were assessed. The null hypotheses were that there would be no difference in 1) interfacial characteristics of the experimental BMEP and a commercial 10-MDP–based system and 2) MMP inhibition of BMEP and a known inhibitor control.

## Materials and Methods

### Reagents

BMEP, 2-hydroxyethyl methacrylate (HEMA), triethyleneglycol dimethacrylate (TEGDMA), camphorquinone (CQ), ethyl 4-(dimethylamino) benzoate (EDAB), and ethanol were purchased from Sigma-Aldrich; Bisphenol A glycidyl dimethacrylate (Bis-GMA) and urethane dimethacrylate (UDMA) from Esschem Europe and Batimastat (BB-94) from Selleck Chemicals, which was then prepared as a 1-mM stock in dimethyl sulfoxide.

### Formulation of Experimental 2-Step Self-Etch Adhesive Systems

#### Primers

Two primers were formulated by mixing 19 wt% of ethanol and water, 1 wt% of CQ and EDAB, with a low and high concentration of BMEP at 15 or 40 wt%, the HEMA content adjusted to 45 or 20 wt%, and designated as BMEP15 and BMEP40, respectively. pH of the primers was measured at room temperature 24 h after formulation using a digital pH meter (Mettler-Toledo Ltd) (Table).

**Table.** pH of the Primers, Chemical Composition, and Instructions for Use of the Adhesive Systems Used in This Study.

Material	Code	pH of the Primers	Composition	Instructions for Use
Clearfil SE Bond 2 (Kuraray, lot number 000071)	CFSE	2.02 ± 0.08	Primer: 10-MDP, HEMA, hydrophilic aliphatic dimethacrylate, CQ, water	1. Apply primer for 20 s2. Gently air-dry for 5 s
				3. Apply adhesive
			Adhesive: 10-MDP, HEMA, bis-GMA, hydrophobic aliphatic dimethacrylate, colloidal silica, CQ, initiators, accelerators	4. Gently air-dry to make a uniform film5. Light-cure for 10 s
Experimental primer and adhesive	BMEP15	1.74 ± 0.07	Primer: BMEP, HEMA, ethanol, water, CQ, EDAB	1. Apply primer actively for 20 s2. Gently air-dry for 5 s
	BMEP40	1.46 ± 0.04	Adhesive: Bis-GMA, UDMA, TEGDMA, HEMA, CQ, EDAB	3. Apply adhesive4. Gently air-dry to make a uniform film5. Light-cure for 10 s

10-MDP, 10-methacryloyloxydecyl dihydrogen phosphate; Bis-GMA, bisphenol A glicidyl dimethacrylate; BMEP, bis[2-(methacryloyloxy) ethyl] phosphate; CQ, camphorquinone; EDAB, ethyl 4-(dimethylamino)benzoate; HEMA, 2-hydroxyethyl methacrylate; TEGDMA, triethyleneglycol dimethacrylate; UDMA, urethane dimethacrylate.

#### Adhesive

A mix of Bis-GMA, UDMA, TEGDMA, HEMA, CQ, and EDAB was used as an experimental adhesive with the tested primers.

**Figure 1.**
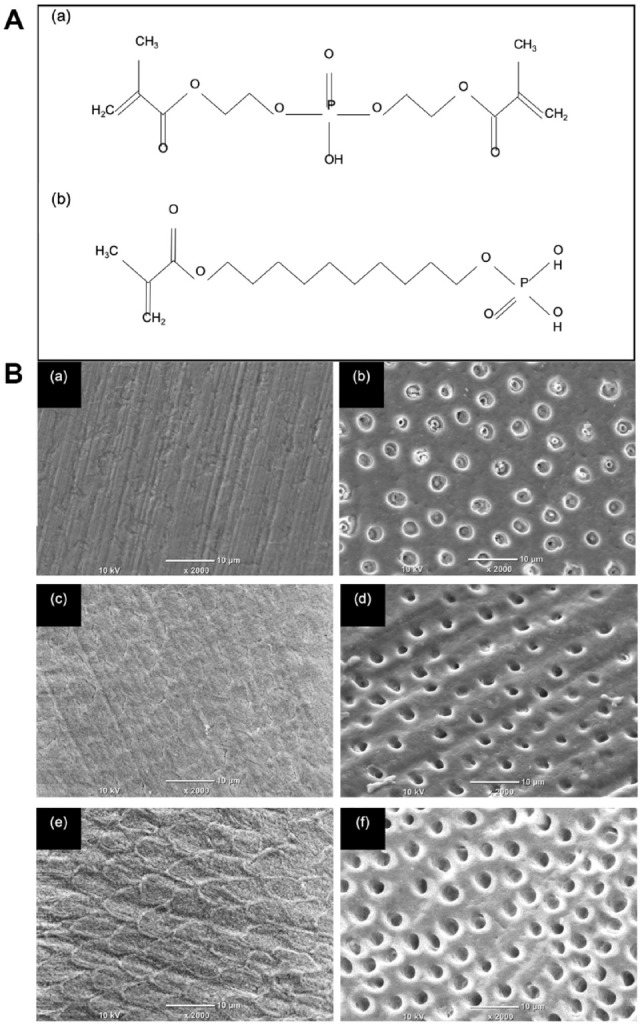
The chemical structure of the phosphoric acid esters used in this study and the etch patterns produced on enamel and dentine. (**A**) The structure of (a) 10-methacryloyloxydecyl dihydrogenphosphate (10-MDP) and (b) bis[2-(methacryloyloxy) ethyl] phosphate (BMEP). (**B**) Scanning electron microscopy images of the self-etch primers on enamel (left column) and dentine (right column) using (a, b) CFSE, (c, d) BMEP15, and (e, f) BMEP40. A distinct etch pattern was obtained with BMEP40 on enamel (e), exposing the enamel prisms. A decrease in pH of the primers (right column, top to bottom) also increased the extent of demineralization, and the dentine tubules were enlarged (f).

Clearfil SE Bond 2 (Kuraray), a 2-step self-etching system, was used as a commercial reference (CFSE). The composition of the primers and adhesive systems is presented in the Table and structures shown in Figure 1A.

### Specimen Preparation

Twenty-three extracted human molars were collected (IRAS ID:157705, REC reference: 16/SW/0220, sponsor: King’s College London). The roots 2 mm below the cemento-enamel junction of each tooth were sectioned using a diamond saw (Labcut 1010; Agar Scientific Ltd) under water cooling. For dentine samples, the occlusal enamel was cut at the junction of the occlusal and middle thirds of the tooth. Samples were prepared by a single, trained operator who performed all laboratory procedures. All teeth were randomly allocated to the treatment groups.

### Scanning Electron Microscopy

Five teeth were sectioned mesiodistally and buccolingually under water cooling to produce 4 sections. Half of these sections had their occlusal surfaces cut to expose dentine. The enamel and dentine sections were then polished wet with 600-grit silicon carbide paper (Struers) and each section treated with 1 of the 3 self-etching primers (*n* = 3 samples per primer per substrate) and rinsed. The teeth were stored for 24 h and sputter-coated with gold. Scanning electron microscopy (SEM) was conducted using Jeol JCM 6000 Plus (JEOL) at an accelerating voltage of 10 kV to analyze etching patterns qualitatively.

### Confocal Laser Scanning Microscopy Interface Evaluation

In total, 0.1% (w/v) rhodamine B and 0.1% (w/v) fluorescein were added to the primers and adhesives, respectively. Nine teeth were primed and bonded with 1 of the 3 SEAs, then restored with a resin composite, UnoDent (Latitude), in two 2-mm increments and polymerized using an LED unit (Elipar DeepCure-S; 3M ESPE) with an output intensity of 1,400 mW/cm^2^ and stored in distilled water for 24 h at 37°C. Subsequently, the teeth were sectioned into 1.2-mm thick sections and polished wet with 1,200-grit silicon carbide paper, and 2 specimens per tooth were randomly selected for confocal laser scanning microscopy (CLSM) (*n* = 6) using a Nikon Ti-E Eclipse A1 inverted confocal laser scanning microscope with a 60×/1.4 NA oil-immersion lens.

### Micro-Raman Spectroscopy

Nine teeth were primed, bonded, restored, and sectioned as described earlier. Line scans of the resin-dentine interface were recorded using a micro-Raman spectrometer (Renishaw) starting from the resin composite toward the dentine at 1-mm intervals (*n* = 3). A water immersion 60×/1.2 NA objective lens Plan Apo VC was used with a 785-nm laser source (25-mW line illumination) and a 600-line/mm diffraction grating. The spectra were Raman-shift-frequency calibrated with known lines of silicon. All spectra were obtained over the spectral region of 700 to 2,000 cm^−1^.

### Fourier-Transform Infrared Spectroscopy

Fourier-transform infrared spectroscopy (FTIR) spectra were obtained using an FTIR spectrometer equipped with an ATR attachment (Spectrum One; Perkin-Elmer). For each of the 3 primers, spectra were obtained after 40-s light polymerization, with and without addition of 5% (w/v) HA powder (Plasma Biotal Ltd.). Self-polymerization of the primer-HA was also evaluated by storing reactants in the dark for 24 h without light initiation.

### Inhibition of rhMMP-2 and rhMMP-8

MMP activity assays were carried out as described elsewhere (Almahdy et al. 2015). Briefly, rhMMP-2 and rhMMP-8 (Sino Biological) were activated using 4-aminophenylmercuric acetate (APMA) and activity assays conducted. The inhibitory effect of neat BMEP, BMEP15, and BMEP40 primers and CFSE primer was evaluated against MMP-2 and MMP-8 using fluorescently quenched gelatin and collagen (Invitrogen), respectively. The 10-MDP–containing primer was included as a monomer control. The assay was performed in a 96-well plate in triplicate for the test groups and an MMP inhibitor control group, using 1 µM BB-94 ((2*R*,3*S*)-*N*^4^-Hydroxy-*N*1-[(1*S*)-2-(methylamino)-2-oxo-1-(phenylmethyl)ethyl]-2-(2-methylpropyl)-3-[(2-thienylthio)methyl]butanediamide). Each well contained 2 μL MMP (19.6 ng/well), 10 μL test compound, 38 μL assay buffer (50 mM Tris, 10 m M CaCl_2_, 150 m M NaCl, 0.005% Brij-35, and 10 µM ZnCl_2_, pH 7.5) and 50 μL substrate solution. The control groups included 1) a positive control: 2 μL MMP and 50 μL substrate solution; 2) an inhibitor control: 2 μL MMP, 10 μL BB-94 (dilution from DMSO stock to 1 µM in assay buffer), and 50 μL substrate solution; 3) a test compound control: 10 μL test compound and 50 μL substrate solution; and 4) a substrate control: 50 μL substrate solution. Kinetic fluorescence measurements were obtained for 60 min at 488/530 nm (Chameleon). Background absorbance was determined from substrate control wells and subtracted from readings containing the FITC-conjugated substrate. The mode of inhibition was then assessed by post hoc addition of 0.01% (w/v) trypsin to all wells. Serial dilutions of BMEP and BMEP15 and BMEP40 primers were prepared from 40% to 0.3% (w/v) in halfway dilutions ×8 to observe the dose-dependent inhibitory effect. The tests were performed in triplicate.

### Statistical Analysis

The inhibitory percentage of rhMMPs was analyzed using 1-way analysis of variance (ANOVA) after testing normality using the Shapiro-Wilk test. Tukey’s post hoc comparison was used to determine differences at a significance level defined at α = 0.05. Statistical analysis was performed using GraphPad Prism software 9.0 for MacOS (GraphPad Software).

## Results

### SEM Evaluation

Figure 1Ba, c, and e illustrates etching patterns on enamel and Figure 1Bb, d, and f on dentine using CFSE, BMEP15, and BMEP40, respectively. The enamel etch pattern was most distinct with BMEP40 while dentine tubules were exposed on application of all 3 self-etching primers, but the extent of demineralization increased with decreasing pH, resulting in an increase in diameter of the dentine tubules.

### CLSM Interface Evaluation

**Figure 2.**
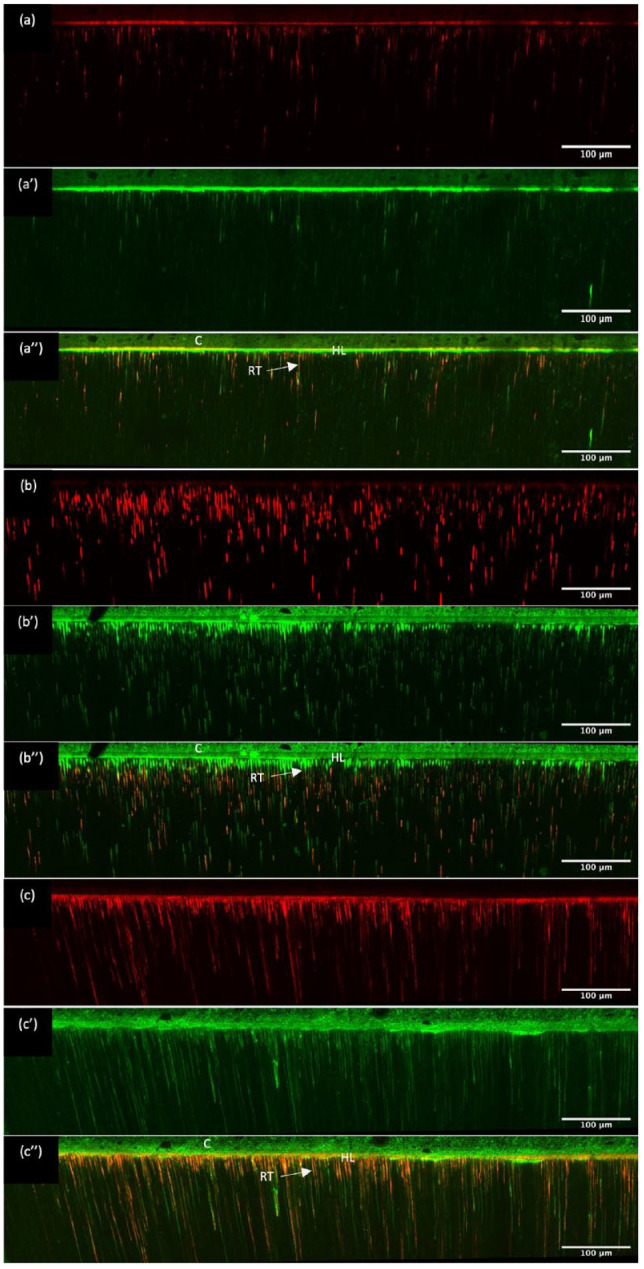
Confocal laser scanning microscopy images showing the hybrid layer formed using the dentine bonding systems. (**A–A′′**) CFSE, (**B–B′′**) BMEP15, and (**C–C′′**) BMEP40. (A–C) Images of the resin-dentine interface where the adhesives were labeled with fluorescein (green). (a′–c′) Images of the resin-dentine interface where the primers were labeled with rhodamine B (red). (a′′–c′′) Composite images demonstrating an orange color, which corresponds to the mixture between the primer and adhesive components, indicating the ability of the dentine bonding systems to diffuse into the etched dentine tubules, creating a gap-free interface and distinct hybrid layer. The images clearly demonstrate the deeper etch pattern created by BMEP15 and BMEP40 primers compared to CFSE, but the corresponding adhesives were able to penetrate the full depth of the etched dentine. C, composite; HL, hybrid layer; RT, resin tags.

Representative CLSM panoramic images of the resin-dentine interface are shown in Figure 2, with all 3 systems exhibiting a resin diffusion zone, forming a clear hybrid layer at the resin-dentine interface with resin tags visible. However, extended resin tags were only observed for BMEP15 and BMEP40 primers with corresponding adhesives penetrating the full depth of etched dentine.

### Micro-Raman Spectroscopy

**Figure 3.**
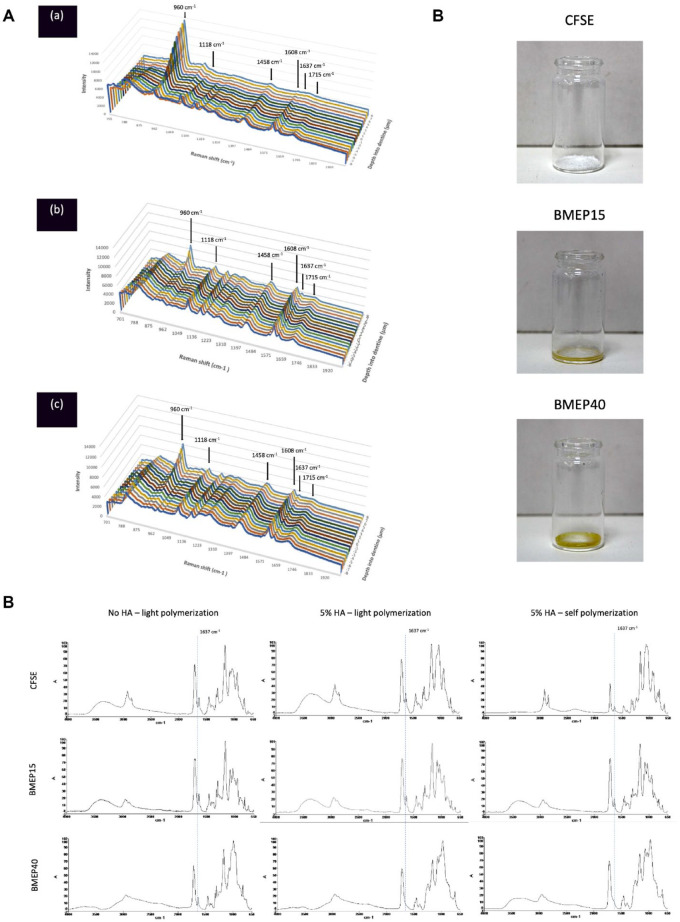
The Raman and FTIR spectra of the primers evaluated in this study as well as the structure of the primers allowed to self-polymerize. (**A**) Raman spectra in the region of 700 to 2,000 cm^−1^ starting from 8 µm into the dentine and ending 8 µm beyond the resin-dentine interface for (a) CFSE, (b) BMEP15, and (c) BMEP40. The characteristic phosphate bands at 960 and 1,118 cm^−1^ are more intense toward the dentine but can still be seen beyond the resin-dentine interface owing to the functional phosphate groups in all bonding systems. The characteristic adhesive bands at 1,458, 1,608, 1,637, and 1,715 cm^−1^ can be detected up to 8 µm into dentine for (b) BMEP15 and (c) BMEP40 groups but not for (a) CFSE, indicating their deeper etching ability. Peak assignments: 1,637 cm^−1^, aliphatic C=C; 1,608 cm^−1^, aromatic C=C; 1,458 cm^−1^, CH_2_; 1,118 cm^−1^, PO_4_ (υ_3_); 960 cm^−1^, PO_4_ (υ_1_). (**B**) Fourier-transform infrared spectroscopy spectra of the primers CFSE, BMEP15, and BMEP40 without hydroxyapatite (HA) following light polymerization (left column), with 5% HA following light polymerization (middle column), and with 5% HA and allowed to self-polymerize in the dark for 24 h (right column). The primer BMEP40 demonstrates complete interaction with HA as demonstrated by the absence of the peak at 1,637 cm^−1^. Peak assignment: 1,637 cm^−1^, aliphatic C=C. (**C**) The structure of the primers left in the dark to self-polymerize following solvent evaporation. CFSE demonstrates the formation of a calcium salt characteristic of 10–methacryloyloxydecyl dihydrogen phosphate (10-MDP)–based systems, BMEP15 demonstrates the formation of a thick consistency, and BMEP40 demonstrates the formation of a solid cross-linked structure.

The Raman imaging line spectra (Fig. 3A) showed characteristic phosphate bands at 960 and 1,118 cm^−1^ arising due to the phosphate groups present in the primers, which increased in intensity on advancing into dentine. The peaks at 1,458 (-CH_2_ stretching), 1,637 (C=C stretching), 1,608 (aromatic C=C), and 1,715 cm^−1^ (-C=O) associated with functional groups in the primer-adhesive system were detected up to 8 µm into dentine for both BMEP15 and BMEP40 but not for CFSE, indicating their deeper ingress. The phosphate peak at 960 cm^−1^ exhibited lower intensity for BMEP15 and BMEP40 in comparison to CFSE due to the greater depth of demineralization.

### FTIR Spectroscopy

FTIR spectra of the primers CFSE, BMEP15, and BMEP40 (Fig. 3B) following light polymerization (left column), with 5% (w/v) HA and light polymerization (middle column) and with 5% HA that was left to self-cure (right column), showed typical stretching vibration bands arising due to carbonyl stretching (1,720 cm^−1^), -CH_2_ stretching (1,428 cm^−1^), aliphatic carbon-carbon double bond (1,637 cm^−1^), and phosphate and hydroxyl groups in the primers as expected. Notably, the peak at 1,637 cm^−1^ (C=C) disappeared in the self-cure BMEP40-HA group with formation of a solid resinous product (Fig. 3C).

### Inhibition of rhMMP-2 and rhMMP-8

**Figure 4.**
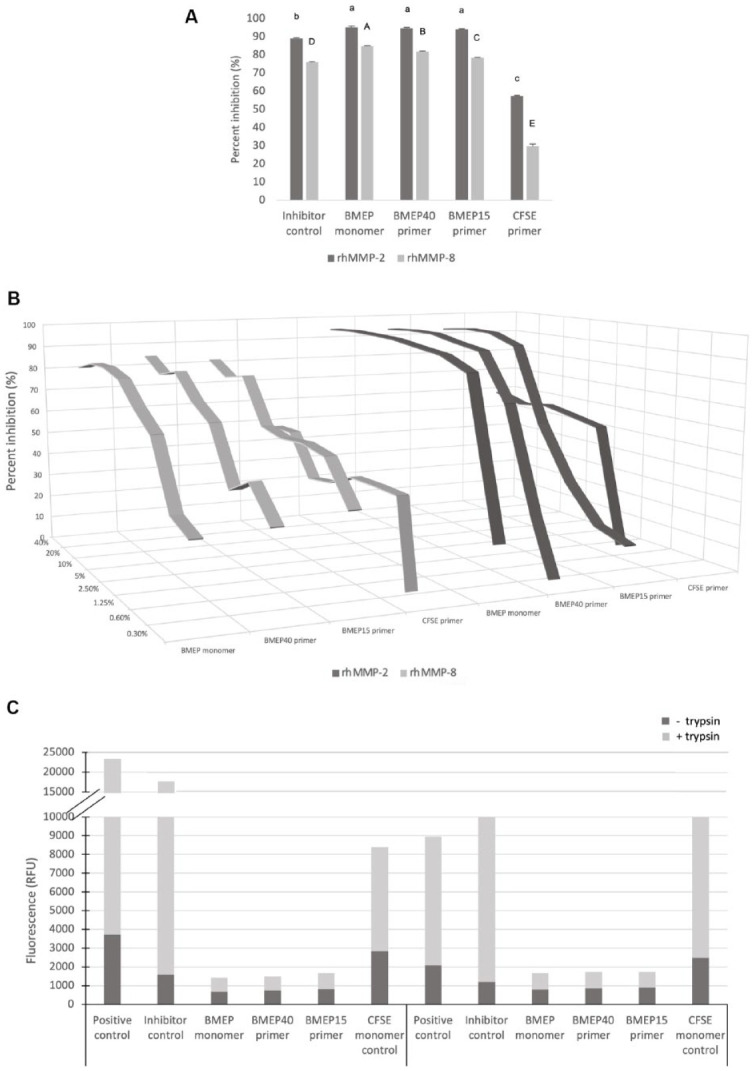
The MMP inhibitory results of the investigated primers, neat BMEP monomer and controls. (**A**) The inhibitory percentage of rhMMP-2 and rhMMP-8 (means and standard deviation) by the test compounds and inhibitor control (BB-94). Similar lowercase and uppercase letters indicate no statistical differences in the percentage of inhibition of rhMMP-2 and rhMMP-8, respectively. (**B**) The dose-dependent inhibitory percentage of rhMMP-2 and rhMMP-8 by the test compounds. (**C**) The mode of inhibition assessed by addition of 0.01% (w/v) trypsin to all wells. Note only partial further cleavage of the gelatin and collagen substrates by trypsin in the BMEP-containing wells, indicating that the substrates were protected by the presence of BMEP.

Figure 4A, B shows the percentage inhibition and dose-dependent inhibitory effect of rhMMP-2 and rhMMP-8, respectively. The inhibitory percentage of rhMMP-2 was significantly higher for BMEP monomer and primers than for the inhibitor control and CFSE (*P* < 0.05), while for rhMMP-8, there was a significant difference among all groups (*P* < 0.05). For rhMMP-2, the percentage inhibition dropped to less than 70% below concentrations of 0.6%, 2.5%, and 5% for the neat BMEP, BMEP40, and BMEP15 primers, respectively. As for rhMMP-8, percentage inhibition dropped to less than 70% below concentrations of 2.5%, 5%, and 10% for the neat BMEP, BMEP40, and BMEP15 primers, respectively. The control monomer did not exhibit MMP inhibition above 70% at any concentration. Figure 4C demonstrates only partial further cleavage of the gelatin and collagen substrates by the trypsin in BMEP-containing wells, a feature not observed with any of the controls.

## Discussion

The success of 2-step adhesive systems depends on etching ability of the primers, which creates a demineralized surface on hard tissues, facilitating the ingress of bonding agents. The ingress of adhesives into enamel governs the bond strength while formation of a hybrid layer in dentine contributes to both strength and long-term integrity of the seal.

The first null hypothesis was rejected since BMEP primers were able to etch both enamel and dentine to a greater extent than CFSE. The enamel etch pattern exposed by BMEP40 most closely resembled that of classic phosphoric acid, while CFSE exhibited much shallower etching with no clear, deep etch pits. Although 10-MDP–based SEAs are capable of conditioning enamel, pre-etching is recommended since a retentive etch pattern is imperative for adequate bonding, which facilitates ingress of bonding agents through diffusion and capillary action, allowing micromechanical interlocking of the resin (Szesz et al. 2016). The absence of smear plugs and precipitate-free surfaces with BMEP primers, unlike CFSE-treated dentine, was attributed to the lower pH, higher hydrophilicity, and better wetting of BMEP primers that enabled concentration-dependent demineralization. The larger diameter of exposed collagen fibrils in BMEP40 compared to BMEP15 under the same vacuum and dehydration conditions is related to pH-dependent solubility of calcium phosphates. Since dentine smear plugs can weaken dentine-resin interfaces (Alshaikh et al. 2018), BMEP primers may have an advantage over CFSE.

The uninterrupted hybrid layer with consistently greater density of etched dentine tubules (Figs. 1B and 2) in BMEP primers is attributed to low pH and higher wettability. Notably, the hydrophobic adhesive traced the BMEP primer within the tubules, suggesting that collagen fibrils were supported through resin tags while cross-linked polymer BMEP networks may render them less susceptible to degradation. The different strategies to minimize effects of hydrolytic and enzymatic degradation of resin-dentine bonds include protection of exposed collagen by cross-linking or entombing with resin, enhancing resistance of naked collagen fibrils through incorporation of MMP inhibitors, remineralization, or combination of these methods. Cross-linking collagen with different agents, such as 1-ethyl-3 (3-dimethylaminopropyl) carbodiimide, acrolein, and glutaraldehyde, or inclusion of MMP inhibitors, such as chlorhexidine, benzalkonium chloride, or proanthocyanidins, has been reported to enhance longevity of resin-dentine bonds (Breschi et al. 2018; Maravic et al. 2018; Mazzoni et al. 2018). However, exogenous cross-linking agents such as glutaraldehyde and acrolein enhance longevity of resin-dentine bonds but are limited by cytotoxicity, while pretreatment adds to the clinical steps.

In contrast to SEA systems where 10-MDP exists in both primer and adhesive to become incorporated within enamel and dentine on etching, the BMEP primer was used in conjunction with a hydrophobic adhesive. The interaction of BMEP primers using Raman imaging spectral data recorded from 8 µm into dentine to 8 µm beyond the resin-dentine interface showed characteristic bands of the phosphate group at 960 and 1,118 cm^−1^. However, the phosphate peak intensity was lower, and carbonyl stretching (1,720 cm^−1^) peaks were also observed at a greater depth with both BMEP15 and BMEP40 at the resin-dentine interface compared to CFSE, indicating greater demineralization and deeper ingress. Similar observations have been reported on model systems of BMEP and EDAB with HA (Zhang and Wang 2012a, 2012b; Liu et al. 2016).

The interaction of BMEP primers with enamel that led to distinct etching, especially at higher concentration, prompted the FTIR analysis of the reaction products of the primers with neat HA to study the self-cure. The spectra revealed typical peaks arising due to the carbonyl group (1,720 cm^−1^), carbon-carbon double bonds (1,637 cm^−1^), methylene scissoring (1,432 cm^−1^), and phosphate groups (1,080, 980 cm^−1^), with intensity of the -C=C- double bond decreasing on light curing. It was interesting to note that BMEP40-HA, when maintained in the dark for 24 h, showed a near-complete monomer conversion, confirming the self-cure polymerization reaction. The reaction product of BMEP40 also yielded a solid resinous polymer while a white flaky precipitate with CFSE confirmed formation of 10-MDP–Ca salts that account for the stable chemical interaction with HA (Fig. 3C). Earlier studies by Liu et al. (2016) also reported a secondary cure of BMEP via chemical initiation, which enhanced degree of cure within similar systems using FTIR and Raman line mapping. Time-resolved FTIR spectra of 10-MDP–HA interaction reported similar findings, the process being dependent on concentration of HA and acidity of the monomer (Liu and Wang 2019). The formation of tertiary amine-acid complexes is known to initiate radical polymerization, but this self-curing is triggered only in presence of a base such as HA when the acidity is high as in BMEP primers. BMEP has a low pH and hence causes deeper demineralization in dentine, which may weaken the dentine-resin interface, but the ability of the adhesive to traverse the depth of etched dentine tubules and entomb dentinal collagen with the cross-linked resin raises the potential for application to both enamel and dentine.

The organic dentine matrix contains endogenous proteolytic enzymes that are responsible for degradation of exposed collagen beneath demineralized hybrid layers (Tjäderhane et al. 2013). BB-94 was previously incorporated into primer and adhesive components of ERAs and SEAs, demonstrating potent MMP inhibition and hence selected as the control inhibitor (Almahdy et al. 2012, 2015). BMEP primers were able to inhibit activity of the abundant dental gelatinases and collagenases (MMP-2 and MMP-8, respectively). The inhibition observed was noncompetitive inhibition in a dose-dependent manner; thus, the second null hypothesis was rejected. The formation of hydrogen bonds between the phosphate group of BMEP and peptide groups in collagen is established (Wu et al. 2016); thus, we expect that the MMP inhibition stearic hindrance resulted in resistance to enzymatic degradation. However, the hydrogen bonding investigated by Wu et al. (2016) considerably decreased after water storage, likely due to hydrolysis of the hydrophilic adhesive used in their study.

Although further work is needed to assess long-term resistance toward hydrolytic and/or collagenolytic degradation, the present findings of using BMEP primers with hydrophobic adhesives exhibit potential. The combination of hydrophilic and hydrophobic moieties into a single component is associated with phase separation and higher susceptibility to hydrolysis (Hashimoto et al. 2003). Past evidence regarding poor performance of hydrophilic adhesives suggests that BMEP may be more suited toward a primer, which can benefit from etching and subsequent inhibition of exposed MMPs. Furthermore, the proposed system avoids potential detrimental effects arising from excessive hydrophilicity by coating the primer with a hydrophobic solvent-free adhesive as presented in this study. Since ERAs still outperform or are similar to SEAs, but with improved marginal integrity, it is likely that longer resin tags, deeper adhesive penetration, and effective etching of enamel can contribute to bond durability (Schroeder et al. 2017; Digole et al. 2020). We propose that this SEA, which combines the high degree of polymerization and collagen-protective potential of BMEP, while minimizing discrepancy between etching and monomer infiltration, may counteract the disadvantages of phosphoric acid etching of ERAs.

In conclusion, the BMEP self-etching primers exhibited good wetting, etching, and penetration in both enamel and dentine. MMP inhibition observed suggests that BMEP may provide additional protection of the resulting bond to gelatinolytic and collagenolytic degradation. Further studies are needed to evaluate bond durability of this experimental system, especially after prolonged water storage, and stability of the resin-encased dentinal collagen using BMEP primer and adhesive systems.

## Author Contributions

R. Alkattan, contributed to conception, design, data acquisition, analysis, and interpretation, drafted and critically revised the manuscript; G. Koller, contributed to data acquisition, analysis, and interpretation, critically revised the manuscript; S. Banerji, contributed to conception and design, critically revised the manuscript; S. Deb, contributed to conception and design, drafted and critically revised the manuscript. All authors gave final approval and agree to be accountable for all aspects of the work.
